# Safety of baricitinib 24 weeks 4 mg or 2 mg for the treatment of rheumatoid arthritis: A meta-analysis of randomized controlled trials

**DOI:** 10.1097/MD.0000000000040512

**Published:** 2024-11-15

**Authors:** Zhihua Shi, Junlong Cai, Ling Yang, Lizhi Tang, Lang She

**Affiliations:** a Department of Pharmacology, Hunan University of Medicine General Hospital, Huaihua, People’s Republic of China.

**Keywords:** baricitinib, JAK inhibitor, meta-analysis, rheumatoid arthritis

## Abstract

**Backgrounds:**

Baricitinib, an oral selective inhibitor of Janus kinase 1 and 2, is approved for moderate and severe rheumatoid arthritis (RA) with insufficient response to conventional synthetic disease-modifying antirheumatic drugs. The study evaluated the safety of baricitinib 24 weeks 4 mg or 2 mg for the treatment of RA.

**Methods:**

The net change (least squares mean [LSM]) of alanine aminotransferase (ALT), creatinine, low-density lipoprotein cholesterol (LDL-C) levels from baseline with the comparison of baricitinib versus placebo was pooled, respectively. The risk ratios (RR) of serious advanced events (SAEs), major cardiovascular events (MACEs), infection, serious infection, and advanced events (AEs) at the end of treatment across groups were compared.

**Results:**

Five randomized controlled trials with 2901 patients were included in the summary analysis. Results showed that baricitinib 4 mg significantly increased ALT and creatinine levels, the net LSM change was respectively 3.59 U/L with 95% confidence interval (CI) (1.75–5.43), 4.25 µmol/L with 95% CI (3.38–5.12), however, baricitinib 2 mg of ALT and creatinine levels were not significantly different. Baricitinib 4 mg and 2 mg significantly increased LDL-C levels, the net LSM change was respectively 11.44 mg/dL with 95% CI (6.08–16.80), 8.70 mg/dL with 95% CI (4.19–13.20). Baricitinib 4 mg significantly increased the incidence of infection, the pooled RR (95% CI) was 1.29 (1.13–1.47), and baricitinib 2 mg was not significantly different. However, the pooled RRs of SAEs, MACEs, and serious infection were not statistically significant across groups. The pooled RRs of AEs were not statistically significant between baricitinib 4 mg and 2 mg.

**Conclusions:**

This study confirmed that patients with RA taking 4 mg baricitinib increased levels of ALT, creatinine, as well as an increased risk of infections, compared with those taking 2 mg baricitinib. Both 2 mg and 4 mg also increased the level of LDL-C, but it increased the most severely at 4 mg baricitinib. However, the incidence of SAEs, MACEs, and serious infection was not significantly different in patients treated with baricitinib 4 mg and 2 mg compared with placebo, the incidence of AEs was not significantly different between baricitinib 4 mg and 2 mg.

## 1. Introduction

Rheumatoid arthritis (RA) is a chronic autoimmune disease that can cause joint swelling and bone erosion, leading to deformity.^[1]^ Conventional synthetic disease-modifying antirheumatic drugs (cDMARDs), such as methotrexate (MTX), have long been the first-line treatment for RA to improve synovitis and physical function. However, many patients do not achieve treatment goals and cannot continue treatment due to severe adverse effects or the development of drug resistance.^[2]^ Therefore, the development of other RA treatment strategies is particularly important. Recent research advances have emphasized the important role of Janus kinase (JAK) inhibitors in the pathogenesis of RA.^[3]^

Baricitinib, a once-daily oral agent that preferentially inhibits JAK1 and JAK2, significantly improves symptoms of moderate-to-severe active RA patients who had undergone an inefficient response or intolerance to cDMARDs.^[4]^ Baricitinib has been approved for the treatment of RA in the European Union, Japan, the USA, and China, which has attracted wide attention.^[5]^ However, considering insufficient clinical safety data, only baricinitib 2 mg but not 4 mg was approved for RA treatment in the USA.^[6]^ Therefore, this study evaluated the safety of baricitinib 4 mg and 2 mg, respectively, compared with placebo. The specific safety concerns included liver and kidney function, blood lipid levels, infection risk, incidence of cardiovascular adverse events, and serious adverse events. The revised FDA application indicates that baritinib 4 mg can be used in patients who have an inadequate response to ≥2 DMARDs or 2 mg.^[7]^

Considering the chronic progressive nature of most RA patients, we wanted to evaluate the safety of different doses of baricitinib for RA. Therefore, we performed a systematic review and meta-analysis of randomized controlled trials (RCTs) evaluating the safety of baricitinib 24 weeks 4 mg and 2 mg in patients with moderate-to-severe RA.

## 2. Methods

We followed the guidelines proposed by the Cochrane Handbook for performing and reporting the current meta-analysis,^[8]^ We conducted this meta-analysis by the PRISMA 2020 checklist.

### 2.1. Search strategy

**Figure 1.**
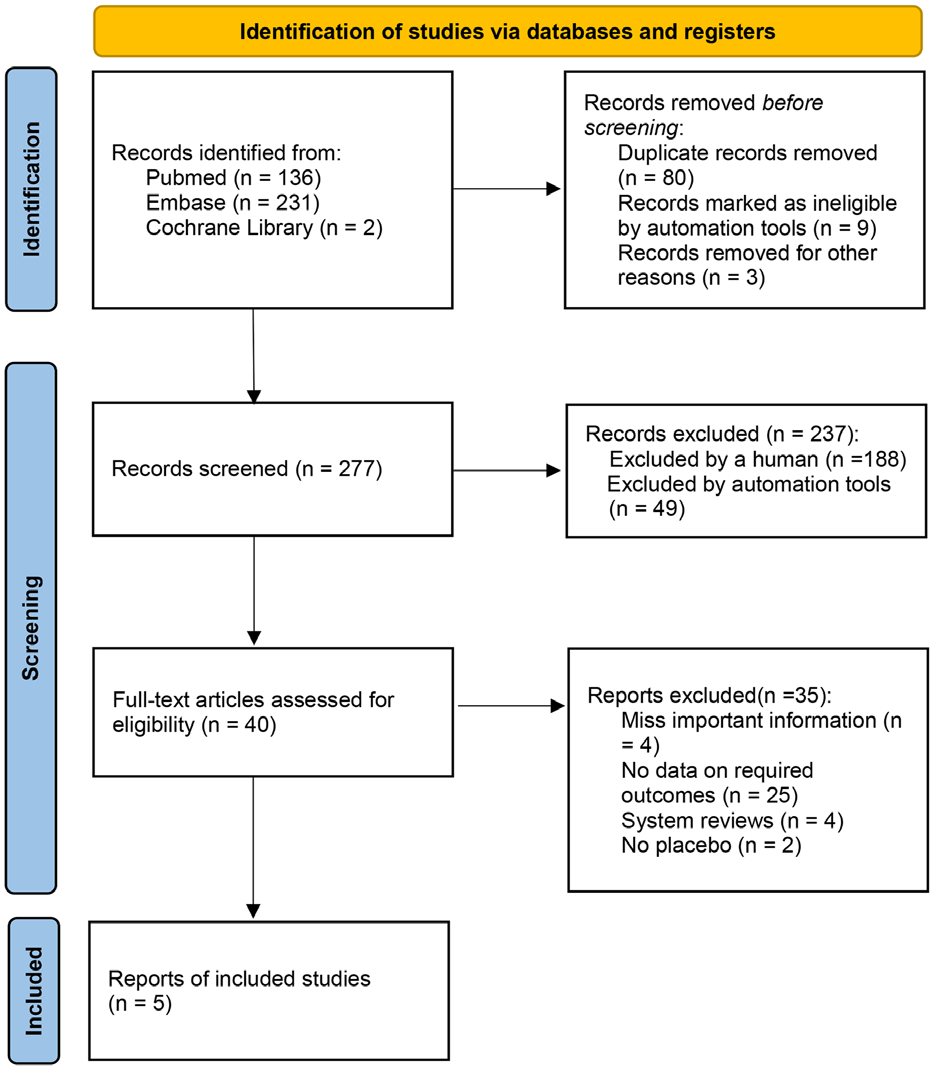
Flow diagram of study selection process.

Studies that reported the safety of baricitinib 24 weeks 4 mg and 2 mg in patients with RA were considered as our interest. Two investigators independently searched the relevant studies across these databases: Pubmed, Embase, and the Cochrane Library (Fig. 1). The following keywords were used for searching: (baricitinib OR LY3009104 OR INCB028050) AND (“rheumatoid arthritis” OR RA). The PubMed search strategy is provided in Supplemental File 1, Supplemental Digital Content, http://links.lww.com/MD/N984. In addition, we also extend the search by scrutinizing the reference lists from all relevant studies or reviews. The last study search was updated on September 1, 2023.

### 2.2. Inclusion and exclusion criteria

The eligible trials were identified by 2 investigators (LS and JLC) independently through a meticulous review of titles, abstracts, and full texts. In the event of any disagreement, consensus was reached through consultation with a third investigator. Original studies were considered eligible if they met the preestablished inclusion criteria: (i) patients were 18 years of age or older and had active RA; (ii) the comparison was the baricitinib vs placebo or active intervention with or without any background therapy; (iii) reported outcomes include the net change of alanine aminotransferase (ALT), creatinine, low-density lipoprotein cholesterol (LDL-C), and the incidence of serious advanced events (SAEs), major cardiovascular events (MACEs), infection, serious infection, and advanced events (AEs) at the end of treatment; (iv) clinical endpoints were provided for the safety of baricitinib 4 mg or 2 mg at 24 weeks. (v) clinical trials of randomized, double-blind, placebo- or active-controlled design. Exclusion criteria: the study included duplicate data; the data could not be extracted or the study did not contain enough data for inclusion; case report, review, basic pharmacological research, animal experiment, etc.

### 2.3. Outcomes

In this study, we focused on the net change of ALT, creatinine, and LDL-C levels at the end of follow-up over baricitinib treatment when compared to the placebo or the active-control (including MTX). The net change was expressed as the least squares mean (LSM) at the end of follow-up from baseline. Net LSM changes were calculated as: (measure at end of follow-up in the treatment group − measure at baseline in the treatment group) − (measure at end of follow-up in the control group − measure at baseline in the control group). LSM refers to the adjusted mean in the main text and is obviously more rigorous since it has been adjusted for potential confounders. In addition, we also calculated the risks of SAEs, MACEs, infections, serious infections versus placebo or active agents, and the incidence of AEs between baricitinib 4 mg or 2 mg.

### 2.4. Data extraction

Two investigators independently extracted the following data from the primary text of individual trial: trial name, publication year, recruitment period, number of centers, patients’ demographics, and clinical characteristics. The proportion of females, duration of RA, randomized patients, background therapy, the net LSM change values from baseline of ALT, creatinine, and LDL-C levels, and the number of patients with severe adverse events, MACEs, infections, serious infections, AEs related to the use of baricitinib, and total numbers of infections related to the use of baricitinib.

### 2.5. Risk of bias

Two investigators (LY and LZT) independently assessed the bias risk and quality of the included trials using the Cochrane Risk of Bias Tool 2.0 and made judgments for each item, such as “high risk,” “low risk,” and “unclear.” According to the guidelines, 5 aspects were assessed: allocation sequence generation, allocation concealment, blinding of participants and investigators, completeness of outcome data, and selective outcome reporting.

### 2.6. Statistical analysis

In this meta-analysis, we compared the net LSM changes of serum ALT, creatinine, and LDL-C levels at the end of treatment from baseline in baricitinib group to those in control group. Pooled effect sizes were represented as weight difference and 95% confidence interval (CI). To estimate the risks of SAEs, MACEs, infections, serious infections, AEs of patients with different treatments were compared and numbers of SAEs, MACEs, infections, serious infections, and AEs in each group were extracted to calculate risk ratios (RRs). To assess the robustness of pooled results, sensitivity analysis was conducted using the leave-one-out method in each turn to investigate the influence of a single study on the overall risk estimate. For the differences in clinical efficacy of different doses of baricitinib, we conducted a subgroup analysis. Between-study heterogeneity was quantitatively assessed using the *I*^2^ index, values of 25%, 50%, and 75% presented as mild, moderate, and high heterogeneity.^[9]^ Potential publication bias was explored by visual inspection of funnel plots for asymmetry.^[10]^

All statistical analyses were performed with Review Manager Version 5.3 (The Nordic Cochrane Center, Copenhagen, Denmark). Statistical significance was judged only for an alpha value of *P* < .05.

### 2.7. Ethical approval

The ethical approval of this study was not necessary, since the included studies are published data and the patients’ privacy was not involved in the design.

## 3. Results

### 3.1. Baseline characteristics of included trials

After a rigorous review of titles, abstracts, and full texts according to prespecified criteria, 5 phase III clinical trials were included in the current meta-analysis, including RA-BUILD, RA-BEGIN, RA-BEACON, RA-BEAM, and RA-BELANCE. All the trials are multicenter studies. Two thousand nine hundred one patients with RA were randomly divided into 3 groups: control group and different doses of baricitinib (2 mg and 4 mg). Seventy-eight percent of patients were female (n = 2285). Except for one trial^[11]^ patients with short RA duration (1.3–1.9 years) who have not received DMARDs treatment, the rest patients have long RA duration (RA duration ≥ 5 years), and have insufficient response or intolerance to ≥1 DMARDs. Most patients (n = 2476) received stable background therapy in addition to placebo or baricitinib, including any DMARD, and 425 patients did not receive any background therapy. The follow-up period was 24 weeks for all studies. More details of study characteristics were presented in Table 1. The meta-analysis of the safety of 4 mg and 2 mg of baritinib was performed at 24 weeks because all data on safety outcomes in the 5 studies were available at that time.

**Table 1:** Study characteristics of included trials.

Trial name	Publication year	Recruitment period	No. of centers	Patients characteristic	Follow-up (weeks)	Randomized patients	Female n (%)	Duration of RA	Background therapy	Control	Doses of baricitinib
RA-BUILD^[12]^	2016	2013.01–2014.05	182	RA patients who had insufficient response or intolerance to ≥1 csDMARD	12–24	684	560 (82)	8	Any DMARDs	Placebo	2 mg and 4 mg, qd
RA-BEGIN^[11]^	2017	2013.01–2014.08	198	RA patients who received no prior DMARDs therapy	24–52	425	304 (72)	1.3–1.9	non	MTX, 10–20 mg, once a week	4 mg, qd
RA-BEACON^[13]^	2016	2013.01–2014.03	178	RA patients who had insufficient response or intolerance to ≥1 csDMARD	12–24	527	431 (82)	14	Any DMARDs	Placebo	2 mg and 4 mg, qd
RA-BEAM^[14]^	2017	2012.11–2014.09	281	Active RA patients had an inadequate response to methotrexate	24–52	975	757 (78)	10	MTX	Placebo	4 mg, qd
RA-BALANCE^[15]^	2020	2014.10–2016.06	30	RA who had an inadequate response to MTX	12–24	290	233 (80)	9.9	MTX	Placebo	4 mg, qd

DMARD = disease-modifying antirheumatic drugs, MTX = methotrexate, RA = rheumatoid arthritis.

### 3.2. Risk of bias

Overall risk of bias was ranked as low in included trials, the other bias was graded as “unclear” in 2 trials (Table 2). It was difficult to correlate the funnel plots used to detect publication bias because the number of studies included in the analysis was too small.

**Table 2:** Risk of bias in the included trials as assessed by the Cochrane risk of bias assessment tool.

	Random sequence generation	Allocation concealment	Blinding of participants and personnel	Blinding of outcome assessment	Incomplete outcome data	Selective outcome reporting	Other bias
RA-BEAM	Low risk	Low risk	Low risk	Low risk	Low risk	Low risk	Low risk
RA-BUILD	Low risk	Low risk	Low risk	Low risk	Low risk	Low risk	Low risk
RA-BEGIN	Low risk	Low risk	Low risk (double-blind)	Low risk	Low risk	Low risk	Unclear
RA-BEACON	Low risk	Low risk	Low risk (double-blind)	Low risk	Low risk	Low risk	Low risk
RA-BALANCE	Low risk	Low risk	Low risk (double-blind)	Low risk	Low risk	Low risk	Unclear

### 3.3. Meta-analysis

#### 3.3.1. Meta-analysis of ALT levels at 24 weeks

**Figure 2.**
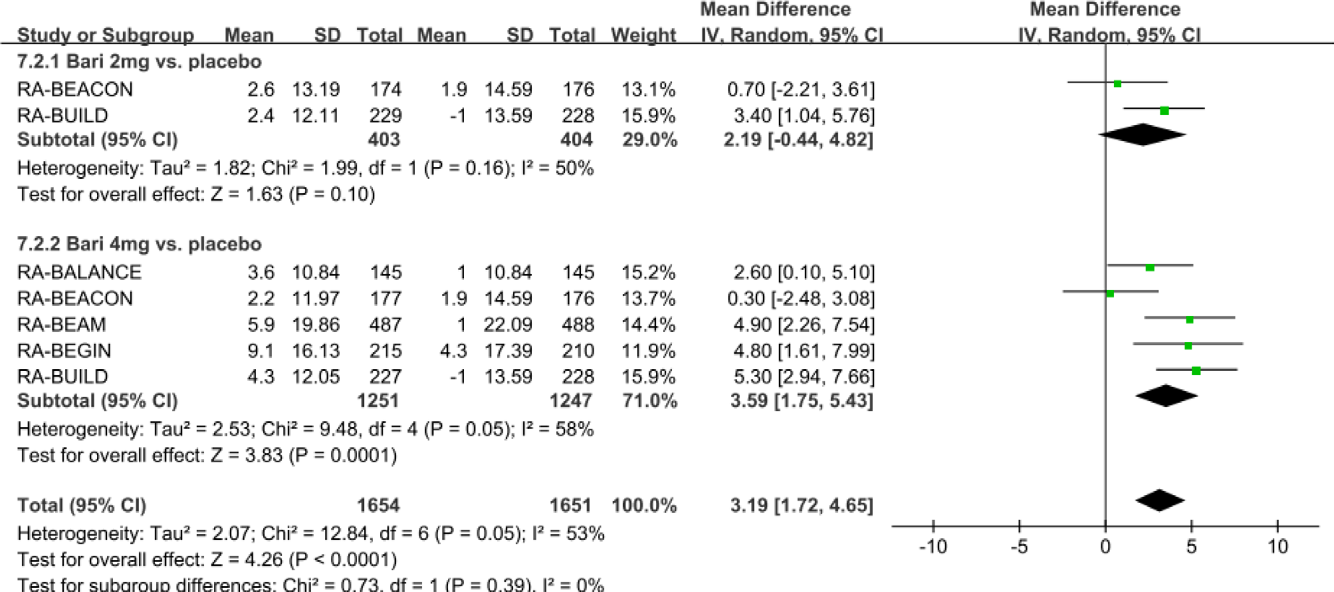
Funnel plot of the effect of baricitinib on ALT levels. Net LSM change of ALT levels were pooled with the comparation of baricitinib 2 mg and 4 mg versus placebo. ALT = alanine aminotransferase, LSM = least squares mean.

The outcomes of this meta-analysis indicated that baricitinib increased the ALT levels, as compared with the placebo group (net LSM change: 3.19 U/L; 95% CI: 1.72–4.65; *P* < .0001; *I*^2^ = 53%) (Fig. 2). Subgroup analysis indicated that baricitinib 4 mg (net LSM change: 3.59 U/L; 95% CI, 1.75–5.43; *P* = .0001; *I*^2^ = 58%) for the treatment of RA significantly increased the level of ALT compared with the placebo group. However, baricitinib 2 mg (net LSM change: 2.19 U/L; 95% CI: ‐0.44–4.82; *P* = .1; *I*^2^ = 50%) has no significant differences in ALT levels, compared with the placebo group.

Sensitivity meta-analysis showed these pooled results of net LSM change of LDL-C were robust with no significant change by any single study.

#### 3.3.2. Meta-analysis of creatinine levels at 24 weeks

**Figure 3.**
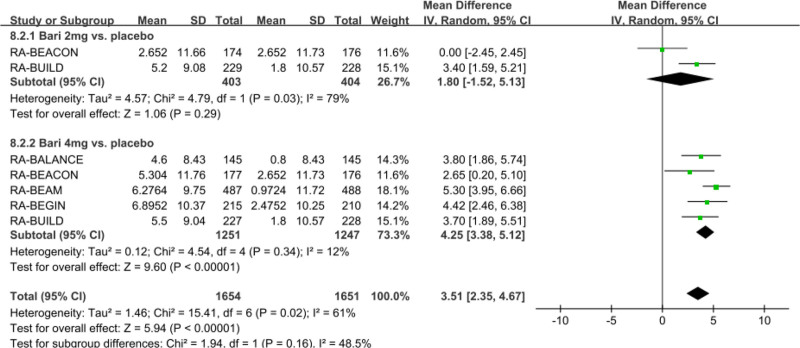
Funnel plot of the effect of baricitinib on creatinine levels. Net LSM change of creatinine levels were pooled with the comparation of baricitinib 2 mg and 4 mg versus placebo. LSM = least squares mean.

Five studies with 2901 patients reported that baricitinib increased the creatinine levels, as compared with the placebo group (net LSM change: 3.51 µmol/L; 95% CI: 2.35–4.67; *P* < .00001; *I*^2^ = 61%) (Fig. 3). Subgroup analysis indicated that baricitinib 4 mg (net LSM change: 4.25 µmol/L; 95% CI, 3.38–5.12; *P* < .00001; *I*^2^ = 12%) for the treatment of RA significantly increased the level of ALT compared with the placebo group. However, baricitinib 2 mg (net LSM change: 1.80 µmol/L; 95% CI: -1.52–5.13; *P* =.29; *I*^2^ = 79%) has no significant differences in creatinine levels, compared with the placebo group.

Sensitivity meta-analysis showed these pooled results of net LSM change of LDL-C were robust with no significant change by any single study, but the heterogeneities have greatly reduced after excluding one study, while the *I*^2^ reduced to 6% from 61% in all doses of baricitinib.

#### 3.3.3. Meta-analysis of LDL-C levels at 24 weeks

**Figure 4.**
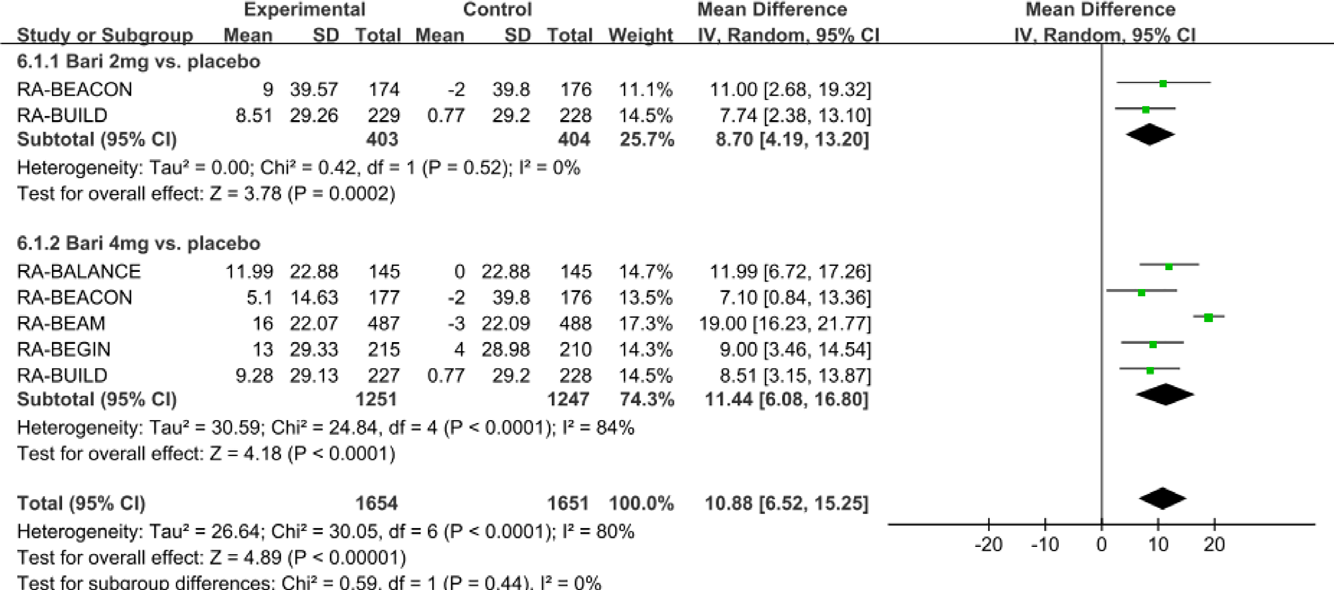
Funnel plot of the effect of baricitinib on LDL-C levels. Net LSM change of LDL-C levels were pooled with the comparation of baricitinib 2 mg and 4 mg versus placebo. LDL-C = low-density lipoprotein cholesterol, LSM = least squares mean.

Five studies with 2901 patients reported that baricitinib significantly increased the LDL-C levels, as compared with the placebo group (net LSM change: 10.88 mg/dL; 95% CI: 6.52–15.25; *P* < .00001; *I*^2^ = 80%) (Fig. 4). Subgroup analysis indicated that both baricitinib 4 mg (net LSM change: 11.44 mg/dL; 95% CI, 6.08–16.80; *P* < .0001; *I*^2^ = 84%) and baricitinib 2 mg (net LSM change: 8.70 mg/dL; 95% CI: 4.19–13.20; *P* = .0002; *I*^2^ = 0%) for the treatment of RA significantly increased the level of LDL-C compared with the placebo group.

Sensitivity meta-analysis showed these pooled results of net LSM change of LDL-C were robust with no significant change by any single study, but the heterogeneities have greatly reduced after excluding one study, while the *I*^2^ reduced to 0 from 80% in all doses of baricitinib and the *I*^2^ reduced to 0 from 84% in baricitinib 4 mg.

#### 3.3.4. Meta-analysis of the incidence of SAEs and MACEs

**Figure 5.**
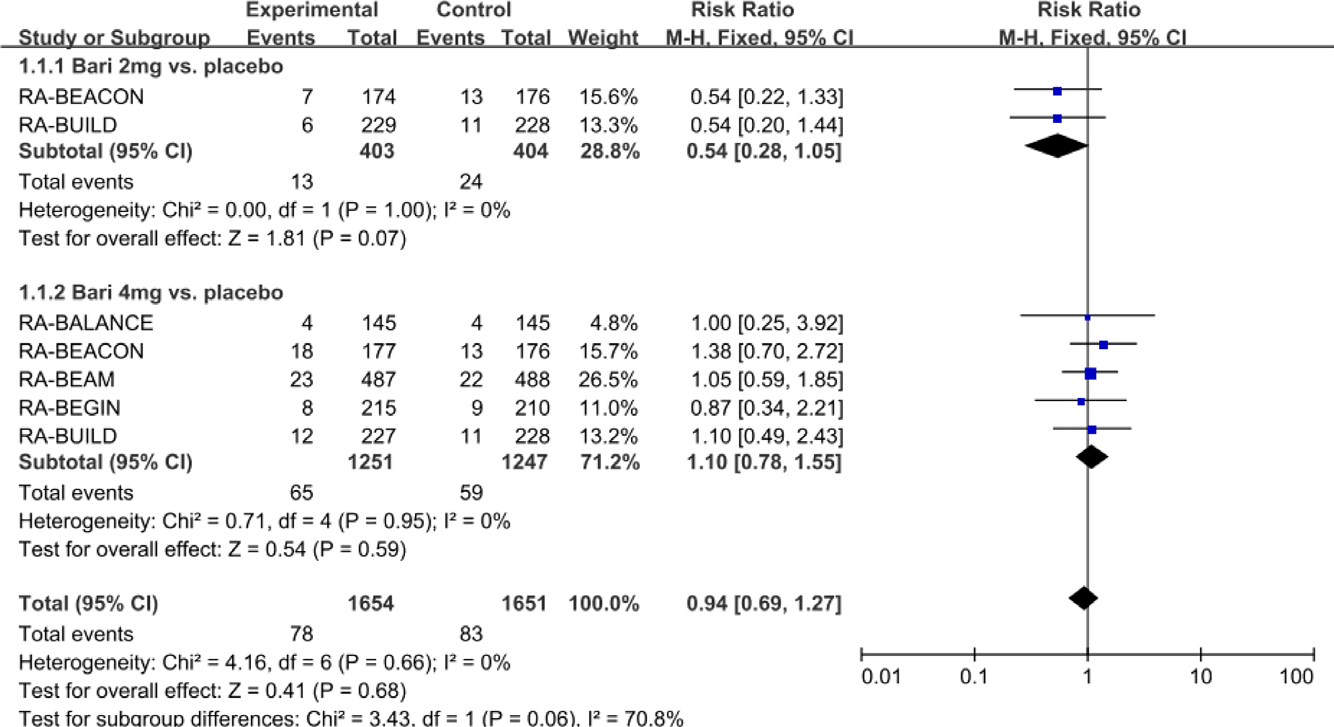
Forest plot for the incidence of serious adverse events.

Five studies with 2901 patients reported the incidence of SAEs in patients with baricitinib 4 mg and 2 mg treatment compared to placebo. As shown in Figure 5, RR was 0.94 (baricitinib vs placebo), 1.10 (baricitinib 4 mg vs placebo), and 0.54 (baricitinib 2 mg vs placebo), but no statistical significance was found across all comparations.

**Figure 6.**
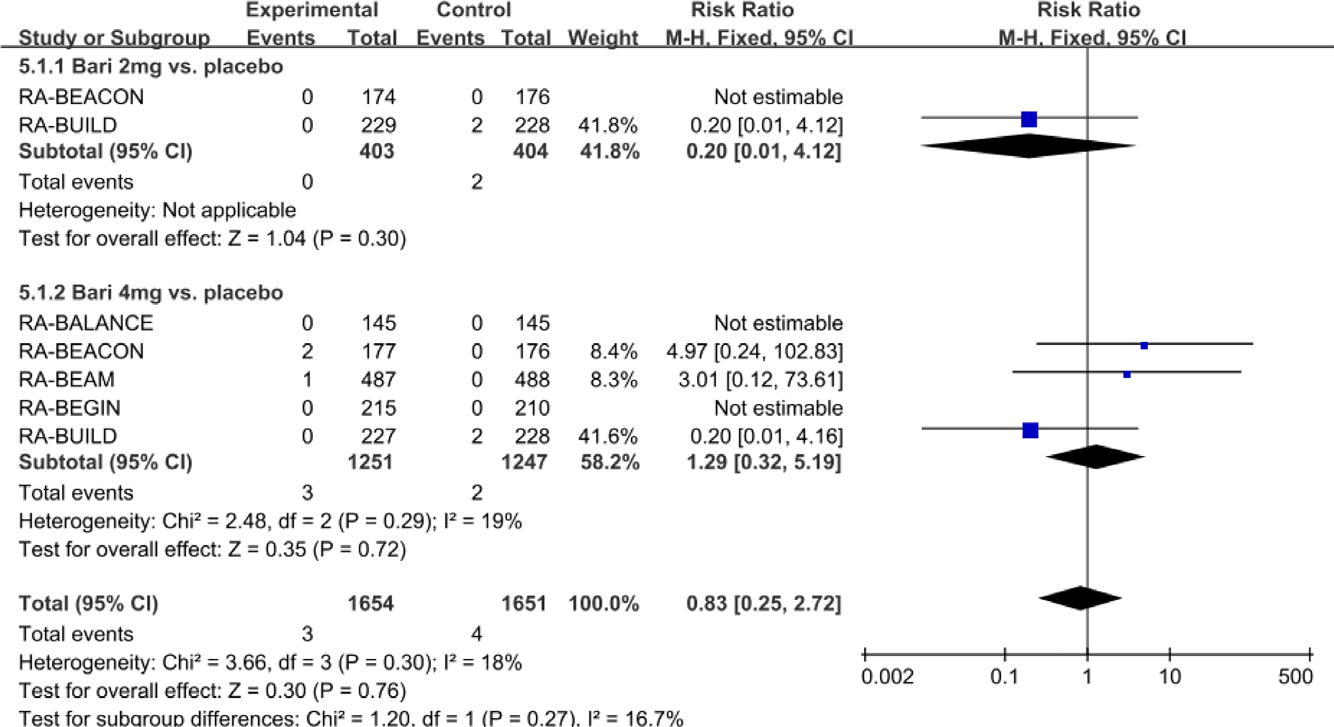
Forest plot for the incidence of major cardiovascular events.

Furthermore, we also observed no significant differences in the incidence of MACEs between all doses of baricitinib and placebo group. As shown in Figure 6, the pooled RR (95% CI) was 0.83(0.25–2.72).

#### 3.3.5. Meta-analysis of the incidence of infections

**Figure 7.**
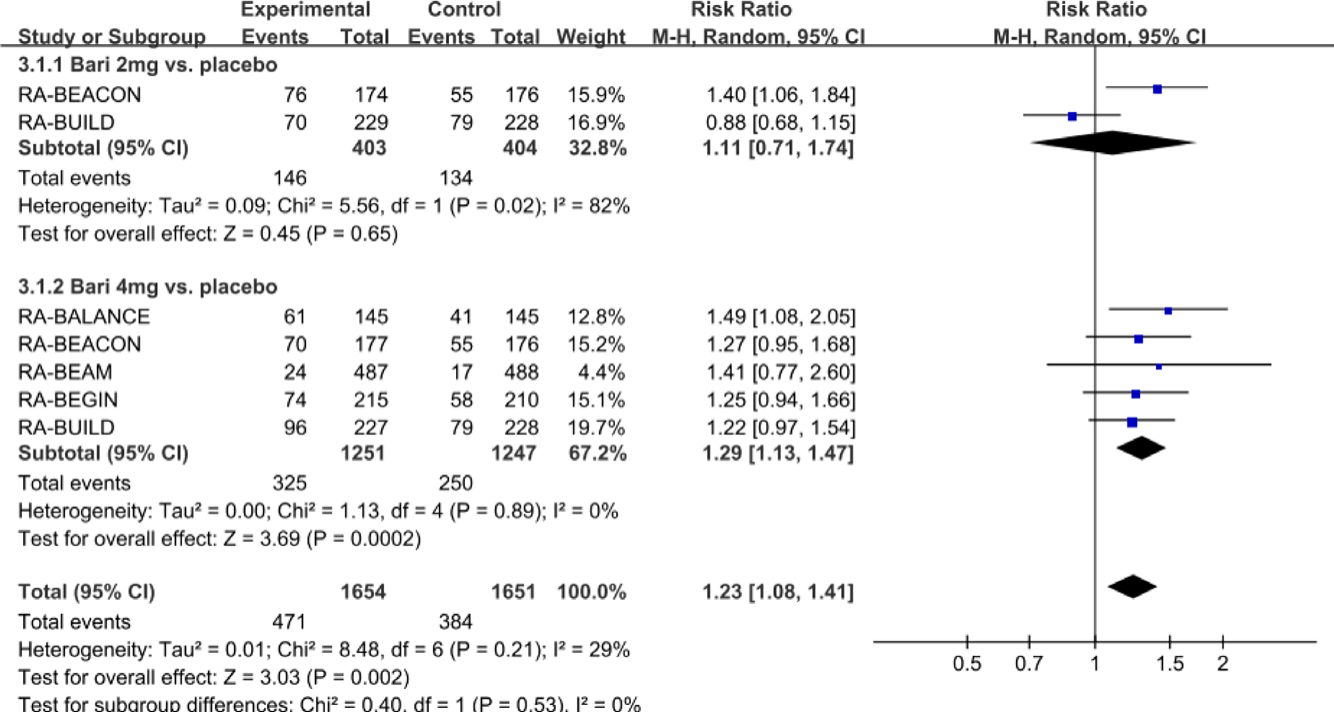
Forest plot for the incidence of infections.

The outcomes of this meta-analysis reported that baricitinib increased the risk of all infections compared to placebo(RR: 1.23; 95% CI: 1.08–1.41; *P* = .002; *I*^2^ = 29%) (Fig. 7). Subgroup analysis indicated that baricitinib 4 mg (RR: 1.29; 95% CI, 1.13–1.47; *P* = .0002; *I*^2^ = 0) for the treatment of RA significantly increased the incidence of all infections compared with the placebo group. However, baricitinib 2 mg (RR: 1.11; 95% CI: 0.71–1.74; *P* = .65; *I*^2^ = 82%) has no significant differences in the incidence of all infections, compared with the placebo group.

#### 3.3.6. Meta-analysis of the incidence of serious infections

**Figure 8.**
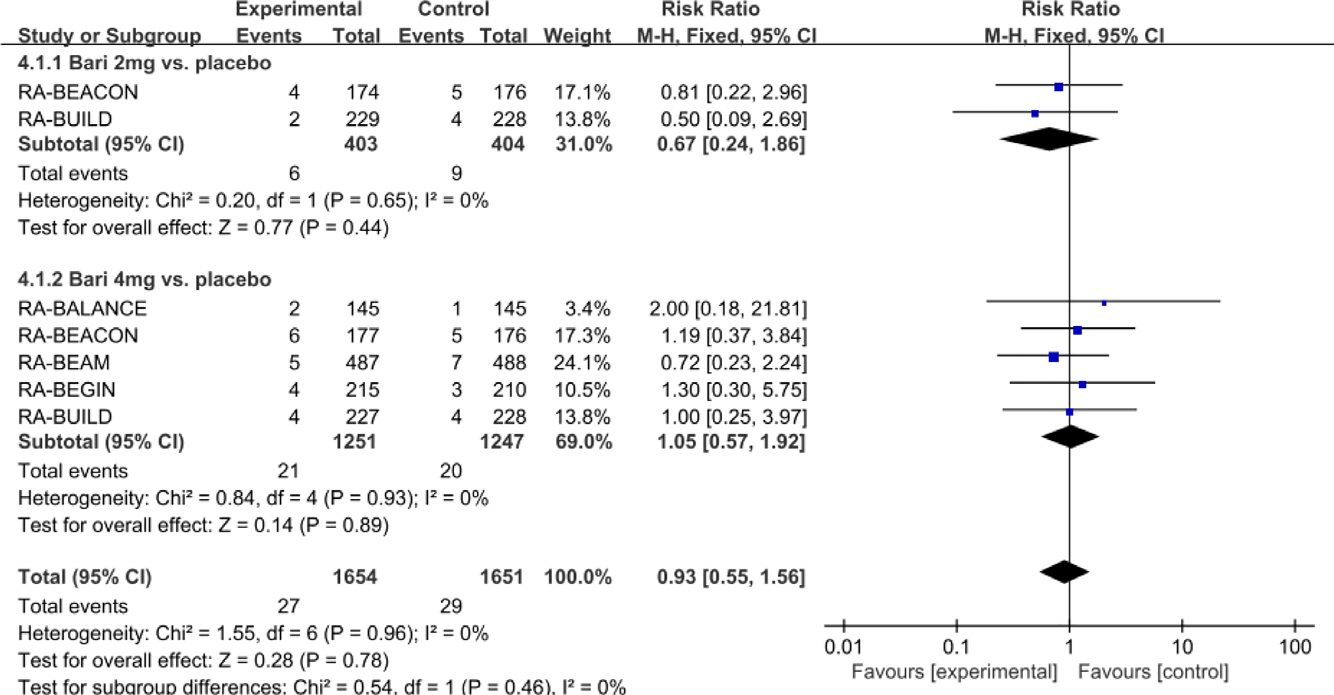
Forest plot for the incidence of serious infections.

Five studies with 2901 patients reported the risk of serious infections in patients with baricitinib 4 mg and 2 mg treatment compared to placebo. As shown in Figure 8, RR (95% CI) was 0.93 (0.55, 1.56) (baricitinib vs placebo), 1.05 (0.57, 1.92) (baricitinib 4 mg vs placebo), and 0.67 (0.24, 1.86) (baricitinib 2 mg vs placebo), but no statistically significant was found across all comparations.

#### 3.3.7. Meta-analysis of the incidence of AEs

**Figure 9.**
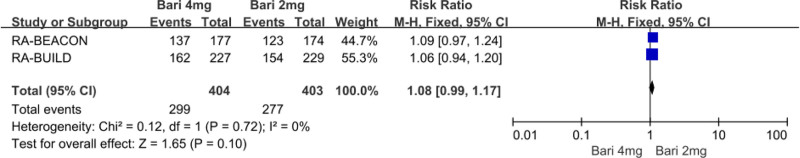
Forest plot for the incidence of adverse events.

Two studies with 807 patients reported that the incidence of AEs in patients with baricitinib 4 mg (RR: 1.08; 95% CI: 0.99–1.17; *P* = .1; *I*^2^ = 0%) has no significant differences, compared to baricitinib 2 mg (Fig. 9).

## 4. Discussion

According to the 2021 American College of Rheumatology Guideline for the Treatment of Rheumatoid Arthritis, JAK inhibitors are recommended if patients have an inadequate response to MTX, other DMARDs, or biologic agents.^[16]^ Patients with selected abnormal laboratory test results and those who had a recent, clinically significant infection were excluded. Patients must have been taking one or more conventional synthetic DMARDs regularly for at least the preceding 12 weeks, with stable doses for at least the preceding 8 weeks. Baricinitib, a selective inhibitor of JAK1 and JAK2, was approved for the treatment of RA in the European Union and Japan in 2017. Baricitinib exhibited 100-fold selectivity for JAK1 and JAK2 over JAK3. The lower affinity for JAK3 could potentially decrease the immunosuppressive effects expected as a consequence of JAK3 inhibition.^[17]^ Baricitinib has an oral bioavailability of approximately 80%. Metabolism is mainly carried out via CYP3A4.^[18]^ The half-life is approximately 12 hours. Approximately 75% of the drug is eliminated via urine and 20% via feces. Of the drugs eliminated, 69% are unchanged in urine and 15% in feces.^[19]^ Given insufficient clinical safety data, only baricinib 2 mg but not 4 mg in the U.S. was approved for the treatment of RA in 2018.^[5,20]^ This meta-analysis, which included 5 RCTs with a total of 2901 RA patients, showed safety data for baricitinib 4 mg and 2 mg at 24 weeks compared with placebo.

The results of this study showed that ALT and creatinine levels increased in RA patients taking 4 mg baricitinib, while there was no significant difference between the 2 groups taking 2 mg baricitinib. Therefore, dose reduction should be considered in RA patients in the presence of liver and kidney failure. Similarly, baricitinib 4 mg and 2 mg both increased the levels of LDL-C in RA patients, but it was most severely increased at baricitinib 4 mg. Interestingly, there was no significant difference in risk for MACEs between baricitinib and placebo. It is possible that the incidence of MACEs was not observed due to the short follow-up duration, but based on these results, we also suggest that dose reduction should be considered in RA patients with hyperlipidemia.

Our meta-analysis did find an increased risk of infection with baricitinib 4 mg compared with placebo. The most common sources of infection are the respiratory and urinary systems. However, there was no significant difference between baricitinib 2 mg and placebo. Interestingly, there was no statistically significant difference in the incidence of serious infections with baricitinib 4 mg compared with placebo. These findings are also consistent with other meta-analyses of JAK inhibitors, suggesting a low incidence of serious infections. Based on these results, baricitinib dose reduction should be considered for RA patients with mild or moderate infections.

Some adverse events may be related to the drug-class, as other JAK inhibitors have similar risks.^[21]^ For example, a large, completed, randomized safety clinical trial showed that tofacitinib increased the risk of thromboembolic events in RA patients.^[18]^ Although baricitinib has not been studied in a large safety clinical trial similar to tofacitinib, the FDA requested that baricitinib, a JAK inhibitor in the same class as tofacitinib, also have a new and updated warning about the risk of thrombosis. In an article in Drug Safety by Scott et al, which used all published data, the estimated thromboembolic risks are approximately 5 events per 1000 patient-years with 4 mg baricitinib daily. Although the baricitinib trial suggested a possible increased risk of thromboembolism, the risk of thromboembolism was significantly increased overall in the RA population. The article concludes that too few events occurred to be certain that they were related and that long-term observational studies are needed to accurately quantify the risk.

The limitations of this study are as follows: first, few literatures were included and the sample size was insufficient. More high-quality, multicenter, and multiregional RCTs are needed for further verification. Second, some safety data need to be observed for a longer period of time, such as the risk of thrombosis and adverse cardiovascular events.

## 5. Conclusions

In summary, baricitinib 4 mg can significantly increase ALT, creatinine, LDL-C levels, and the risk of infections; therefore, dose reduction is considered for patients with hepatic and renal insufficiency, hyperlipidemia, and mild to moderate severity infections. However, the incidence of SAEs, MACEs, and serious infection were not significantly different in patients treated with baricitinib compared with placebo. The incidence of AEs was not significantly different between baricitinib 4 mg and 2 mg. Due to the limitations of this study, more long-term, multicenter, and high-quality studies are needed to further verify the conclusion.

## Acknowledgments

The authors thank the Hunan University of Medicine General Hospital for the technical guidance.

## Author contributions

**Conceptualization:** Lang She.

**Data curation:** Junlong Cai, Ling Yang, Lang She.

**Formal analysis:** Junlong Cai, Ling Yang.

**Investigation:** Lizhi Tang, Zhihua Shi.

**Methodology:** Lizhi Tang, Zhihua Shi.

**Writing – original draft:** Lang She, Zhihua Shi.

**Writing – review & editing:** Lang She, Zhihua Shi.

## Supplementary Material
