# Supplementary material for: Safety of baricitinib 24 weeks 4 mg or 2 mg for the treatment of rheumatoid arthritis: A meta-analysis of randomized controlled trials
Source: Medicine (Baltimore). 2024 Nov 15;103(46):e40512. doi: 10.1097/MD.0000000000040512 (PMC11576007; doi:10.1097/MD.0000000000040512)
Supplement: Supplementary file 1 [file medi-103-e40512-s001.docx]

**Additional file 1.** Search algorithm of Medline

| PubMed (Medline) | | |
| --- | --- | --- |
| #1 | baricitinib OR LY3009104 OR INCB028050 | 1357 |
| #2 | "rheumatoid arthritis "OR RA | 218300 |
| #3 | #1 AND #2 | 460 |
| #4 | randomized controlled trial[Publication Type] | 600409 |
| #5 | controlled clinical trial[Publication Type] | 690914 |
| #6 | randomized [tiab] | 672158 |
| #7 | randomly [tiab] | 416573 |
| #8 | trial [tiab] | 779439 |
| #9 | #4 OR #5 OR #6 OR #7 OR #8 | 1636890 |
| #10 | (animals [mh] NOT humans [mh]) | 5149927 |
| #11 | #3 AND #9 | 136 |
| #12 | #11 NOT #10 | 136 |
